# MicroRNA-21 Promotes the Viability, Proliferation and Milk Fat Synthesis of Ovine Mammary Epithelial Cells by Targeting *PDCD4*

**DOI:** 10.3390/ijms26041460

**Published:** 2025-02-10

**Authors:** Liyan Hu, Jiqing Wang, Zhiyun Hao, Xian Guo, Mingna Li, Xinmiao Wu, Huimin Zhen, Chunyan Ren, Yuan Zhao, Pan Yang, Xuanyu Wang

**Affiliations:** 1 College of Animal Science and Technology, Gansu Agricultural University, Lanzhou 730070, China; huliyan2020@163.com (L.H.); haozy@gsau.edu.can (Z.H.); limn@gsau.edu.cn (M.L.); wuxinmiao2020@163.com (X.W.); zhenhm@st.gsau.edu.cn (H.Z.); renyaya86@126.com (C.R.); 18709405439@163.com (Y.Z.); 15709319461@163.com (P.Y.); gsauwxy@163.com (X.W.); 2 Lanzhou Institute of Husbandry and Pharmaceutical Sciences, Chinese Academy of Agricultural Sciences, Lanzhou 730050, China; guoxian@caas.cn

**Keywords:** miR-21, ovine mammary epithelial cells, *PDCD4*, activity, triglyceride

## Abstract

MicroRNAs (miRNAs) are short endogenous non-coding RNAs and play important roles in regulating mammary development and activities of ovine mammary epithelial cells (OMECs), which affect the milk yield and milk ingredient contents of ewes. We previously found that miR-21 was highly expressed in ovine mammary tissue, while the regulatory mechanisms of miR-21 underlying mammary development and lactation performance are still unclear. Accordingly, in this study, we investigated the functions of miR-21 in the activities of OMECs, and validated the target relationship of miR-21 with a predicted target gene programmed cell death 4 (*PDCD4*) by a dual-luciferase reporter assay. Finally, we investigated the regulatory effect of *PDCD4* on the viability, proliferation and milk fat synthesis of OMECs. The overexpression of miR-21 significantly increased the viability of OMECs, the number and proportion of Edu-labeled positive OMECs, as well as the contents of triglyceride in OMECs. In fact, miR-21 inhibitor obtained opposite results with miR-21 mimics. The results obtained from the dual luciferase report and RT-qPCR assays confirmed that the seed sequence of miR-21 can complementarily combine with the 3′-untranslated regions (3′-UTR) of *PDCD4*, and miR-21 decreased the luciferase activity of *PDCD4*. Meanwhile, miR-21 also reduced the expression of *PDCD4*. These results indicate that *PDCD4* is a target gene of miR-21. It was further found that *PDCD4* decreased the viability and triglyceride content of OMECs, and the number and proportion of Edu-labeled positive OMECs. These findings suggest that miR-21 promotes the viability, proliferation and milk fat synthesis of OMECs by down-regulating the expression of *PDCD4*. The results revealed the regulatory mechanism by which miR-21 affected the activities and milk fat synthesis of OMECs in sheep.

## 1. Introduction

Lactation performance is an essential component of the economic traits of livestock animals. It has a significant impact on the production performance of offspring and is also an important indicator by which to measure genetic values [1]. The lactation performance of ewes mainly depends on milk yield and milk composition. Milk is known to be synthesized by the absorption of nutrients from the blood by mammary epithelial cells (MECs), and their number and activities therefore affect the yield and quality of milk [2,3]. In this context, the investigation of the regulatory mechanisms underlying the number and activity of MECs is crucial for improving the lactation performance of ewes.

MicroRNAs (miRNAs) are endogenous non-coding RNAs that widely participate in various biological activities, with a length of approximately 18–21 nucleotides [4]. miRNAs are known to play roles in organisms mainly through complementary pairing with the 3′-UTR of the target mRNAs, thereby inhibiting the translation or promoting the degradation of the target genes at a post-transcriptional level [5,6]. It has been indicated that miRNAs are widely involved in development and lactation of the mammary gland, as well as the synthesis and metabolism of milk components [7,8,9]. Previously, Nagaoka et al. [7] revealed that miR-200a inhibited the differentiation of MECs by affecting their cavity formation rate and polarity. Lin et al. [8] found that the overexpression of miR-103 increased the expression of genes related to milk fat synthesis, thereby promoting the formation of fat droplets, and the accumulation of triglycerides and unsaturated fatty acids. miR-21-3p has been reported to promote the formation of triglycerides in bovine MECs by targeting long-chain fatty acid elongase 5 (*Elovl5*) [9]. In spite of this, the regulatory mechanisms of many miRNAs underpinning lactation performance and mammary development remain unclear.

miR-21 is located on chromosome 11 of the sheep genome and is widely expressed in various tissues, such as the heart, liver, spleen, mammary gland and skin, eventually participating in various biological activities of the body [10]. Increasing numbers of reports have indicated that miR-21 is widely involved in the regulation of physiological and pathological activities of mammary gland tissue [11,12]. For example, Feuermann et al. [13] found that miR-21 was indispensable for mammary gland development and lactation in mice. A study by Zhu et al. [14] showed that miR-21 plays a role in cell invasion and tumor metastasis in metastatic breast cancer MDA-MB-231 cells by targeting multiple tumor/metastasis suppressor genes. miR-21-3p was up-regulated in the expression in high-fat breast tissue, indicating that miR-21 may be responsible for lipid synthesis [15,16]. However, it is still unclear whether miR-21 has a regulatory effect on the activities and milk fat synthesis of OMECs.

*PDCD4* is a tumor suppressor gene induced during programmed cell death, which plays a role in cell apoptosis and DNA damage response [17,18]. Recent studies have found that miR-21 can regulate various cell proliferation and apoptosis processes by binding to the 3′-UTR regions of *PDCD4*. For example, miR-21-5p inhibited the phosphorylation of serine/threonine kinase PKB (AKT) by targeting *PDCD4*, thereby inhibiting the apoptosis of porcine endometrial epithelial cells [19]. miR-21 negatively regulated the expression level of *PDCD4* in renal cell carcinoma and then increased the invasiveness and metastasis of cancer cells [20]. In our previous study, miR-21 was found to be highly expressed using RNA-seq in ovine mammary gland tissue [21]. So far, it is still unknown whether miR-21 can play a role in the ovine mammary gland development and lactation process by targeting *PDCD4* in sheep. Accordingly, in this study, we validated the target relationship between miR-21 and *PDCD4*. We also analyzed the effects of miR-21 and *PDCD4* on the viability, proliferation and triglyceride content of OMECs, with a clear aim to elucidate the molecular mechanism of miR-21 regulating lactation performance in sheep.

## 2. Results

### 2.1. Detection of Transfection Efficiency of miR-21 and PDCD4 into OMECs

**Figure 1:**
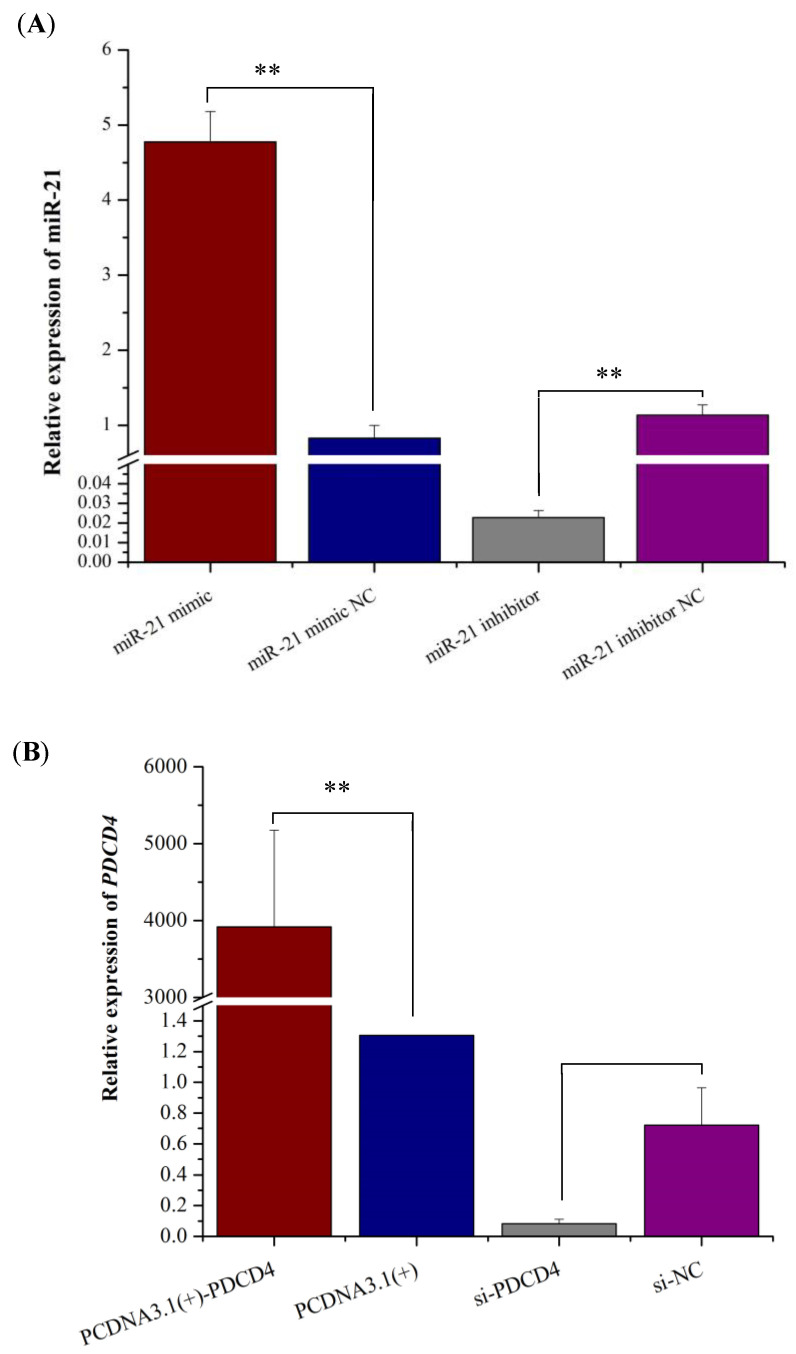
The detection of transfection efficiency of miR-21 and *PDCD4* into ovine mammary epithelial cells (OMECs). (**A**) The expression levels of miR-21 when miR-21 mimic and miR-21 inhibitor were transfected into OMECs. (**B**) The expression levels of PDCD4 when pcDNA 3.1(+)-PDCD4 and si-PDCD4 were transfected into OMECs. ** *p* < 0.01.

The RT-qPCR analysis showed that compared with the NC group, miR-21 mimic significantly increased the expression level of miR-21 in OMECs (Figure 1A), while interference with miR-21 inhibitor significantly reduced its expression level (Figure 1A). These indicate that miR-21 mimic and miR-21 inhibitor have been successfully transfected into ovine OMECs and can be used for subsequent experiments.

The relative expression level of *PDCD4* significantly increased in cells transfected with pcDNA3.1(+)-*PDCD4* when compared to the pcDNA3.1(+) group (*p* < 0.01, Figure 1B). On the contrary, si-*PDCD4* led to a significantly decrease in expression of *PDCD4* compared with its NC group (*p* < 0.01, Figure 1B).

### 2.2. The miR-21 Promotes the Proliferation and Viability of OMECs

**Figure 2:**
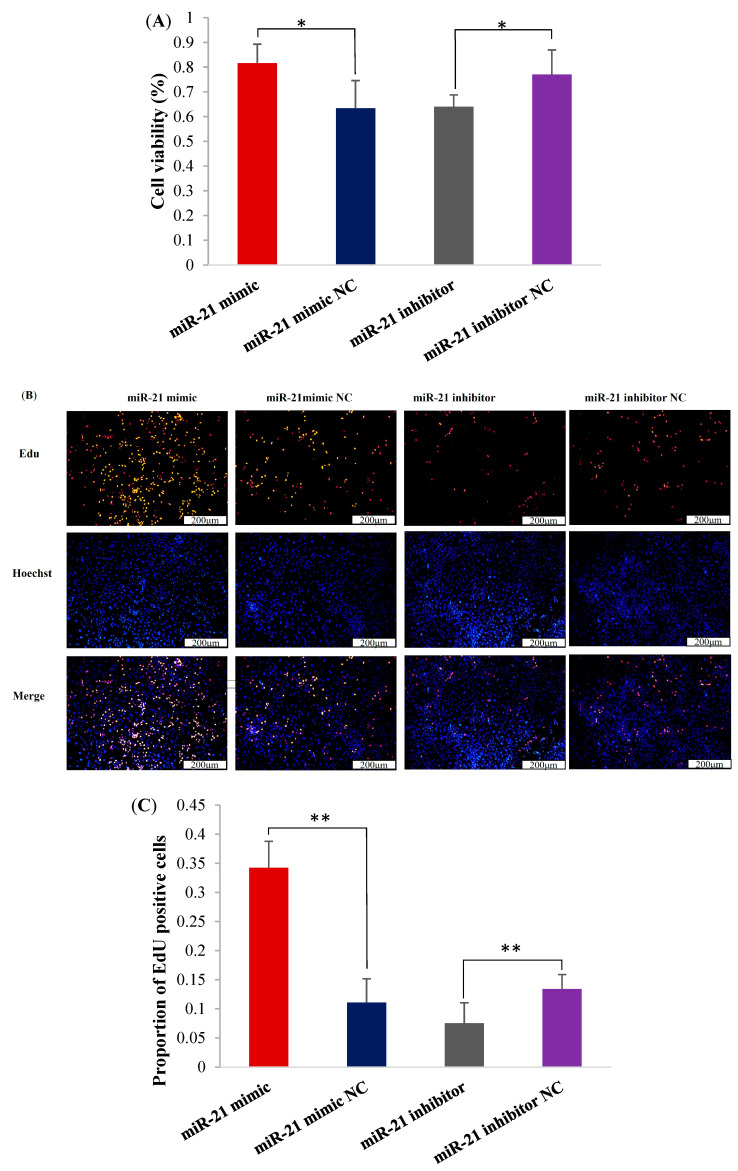
miR-21 promoted the proliferation and viability of ovine mammary epithelial cells (OMECs). (**A**) The viability of OMECs detected after transfection with miR-21 mimic and miR-21 inhibitor. (**B**) The Edu assay for detecting the effect of miR-21 on the proliferation of OMECs. (**C**) The proportion of Edu-labeled positive OMECs. * *p* < 0.05 and ** *p* < 0.01.

The cell counting kit-8 (CCK8) assay and 5-Ethynyl-2′-deoxyuridine (EdU) assay analysis results showed that the overexpression of miR-21 significantly increased the viability of OMECs (Figure 2A), as well as the number (Figure 2B) and proportion (Figure 2C) of Edu-labeled positive OMECs compared with its NC group, while miR-21 inhibitor markedly reduced the viability of OMECs, and the number and proportion of Edu-labeled positive OMECs (Figure 2).

### 2.3. The miR-21 Increases Triglyceride Content

**Figure 3:**
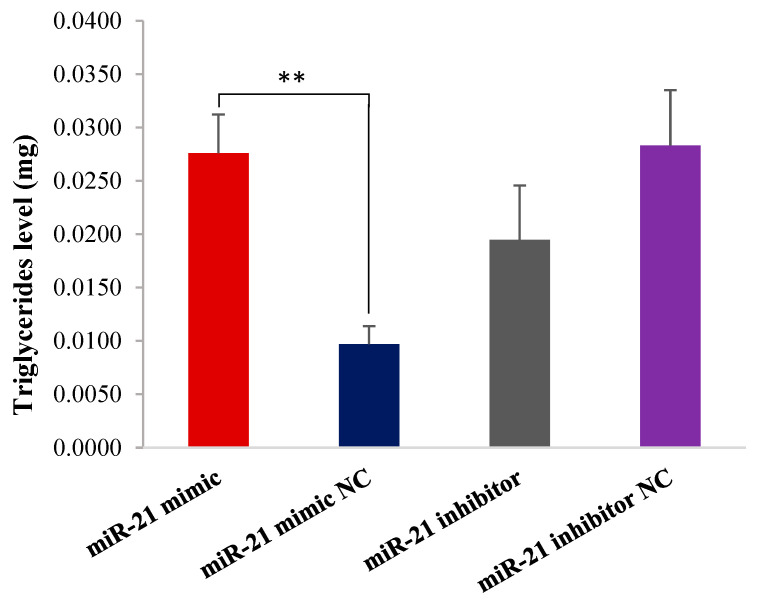
The triglyceride level of ovine mammary epithelial cells (OMECs) transfected with miR-21 mimic and miR-21 inhibitor. ** *p* < 0.01.

Compared with the miR-21 mimic NC group, miR-21 mimic significantly increased the triglyceride content in OMECs (*p* < 0.01, Figure 3). However, there was no significant difference in triglyceride content between miR-21 inhibitor and miR-21 inhibitor NC (*p* > 0.05, Figure 3). These results indicate that miR-21 promotes triglyceride synthesis of OMECs.

### 2.4. PDCD4 Is a Target Gene of miR-21

**Figure 4:**
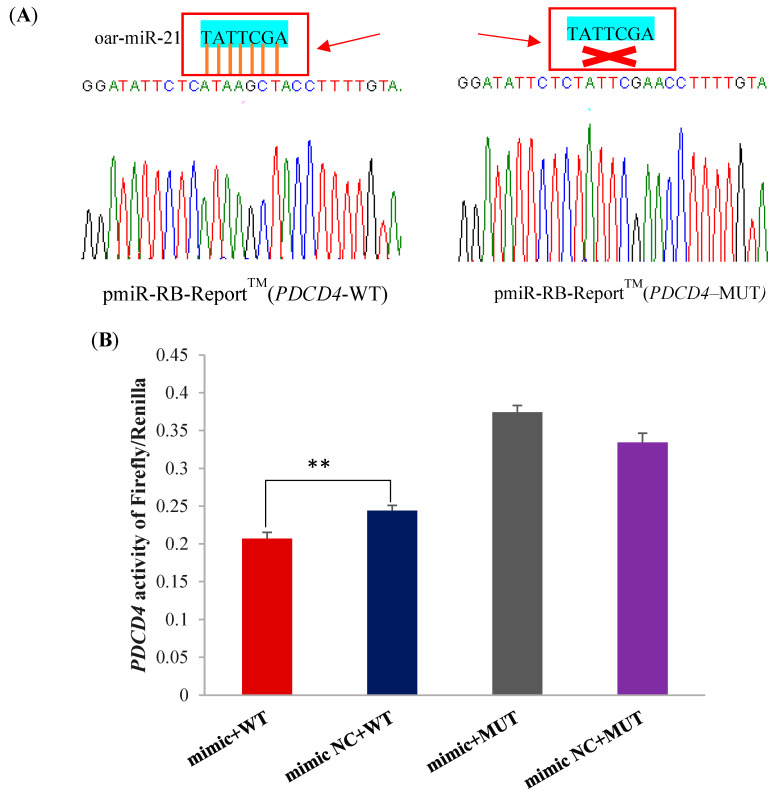
The validation of the target relationship between miR-21 and *PDCD4* using a dual luciferase report assay. (**A**) The validation of dual luciferase report vectors using Sanger sequencing. (**B**) The luciferase activity detection of *PDCD4* when miR-21 mimic or miR-21 mimic NC, and wild-type or mutate-type luciferase reporter vectors were co-transfected into HEK293T cells. ** *p* < 0.01.

In order to verify the target relationship between miR-21 and its predicted target gene *PDCD4*, the dual luciferase reporter vectors were constructed and confirmed using Sanger sequencing (Figure 4A). The miR-21 mimic significantly decreased the luciferase activity of *PDCD4* in the wild-type dual luciferase vector (Figure 4B). However, miR-21 mimic did not significantly affect the activity of *PDCD4* in the mutate-type dual luciferase vector (Figure 4B). Taken together, these results indicate that *PDCD4* is a target gene of miR-21.

### 2.5. miR-21 Down-Regulates the Expression of PDCD4

**Figure 5:**
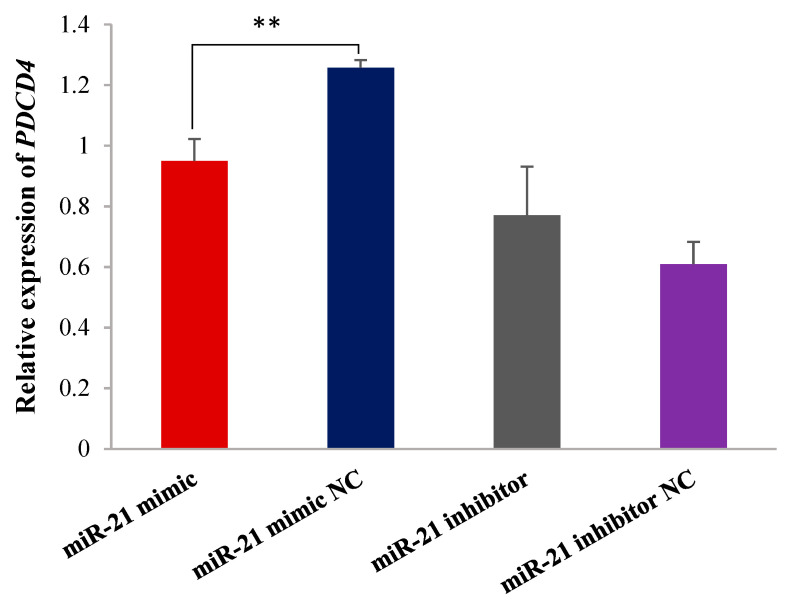
miR-21 negatively regulated the expression of *PDCD4*. ** *p* < 0.01.

Subsequently, we transfected miR-21 mimic and miR-21 inhibitor into OMECs to evaluate whether miR-21 affected the expression of *PDCD4*. The results showed that miR-21 mimic significantly reduced the expression level of *PDCD4* in OMECs (*p* < 0.01), while miR-21 inhibitor obtained the opposite result with miR-21 mimic (Figure 5). These results indicate that miR-21 down-regulated the expression of *PDCD4* at the post-transcriptional level.

### 2.6. PDCD4 Inhibits Proliferation and Viability of OMECs

**Figure 6:**
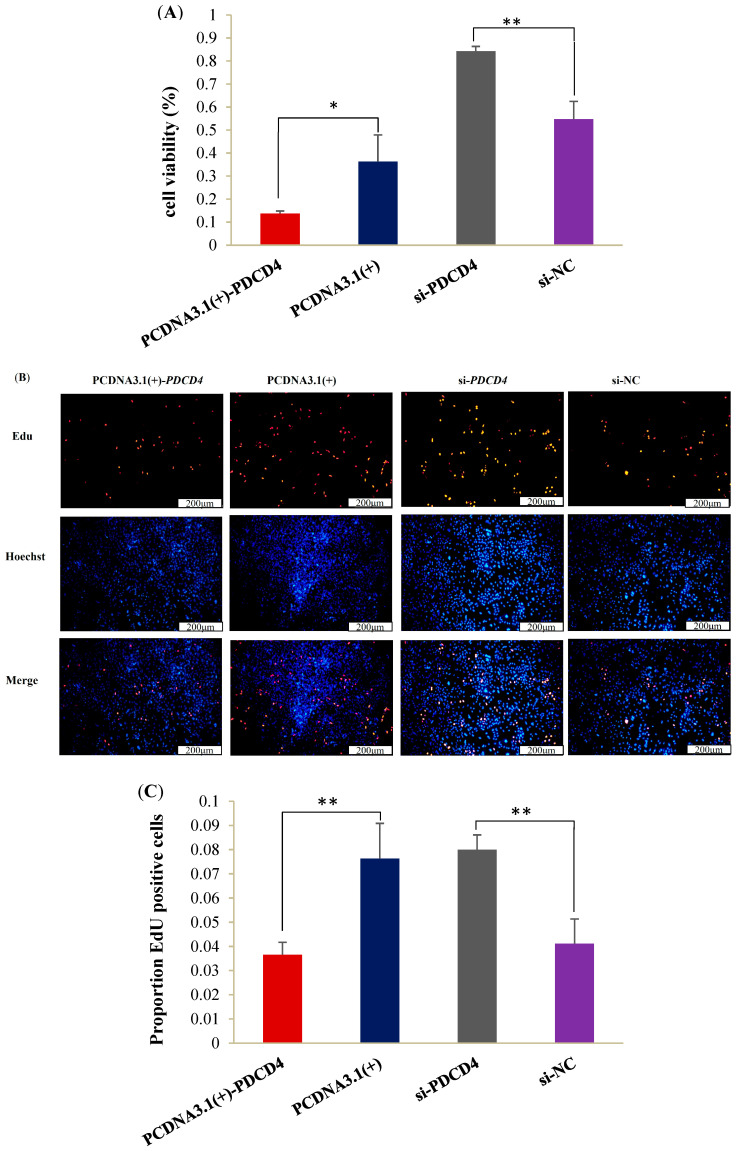
The *PDCD4* inhibited the viability and proliferation of ovine mammary epithelial cells (OMECs). (**A**) The viability of OMECs after transfection with pcDNA3.1(+)-*PDCD4* and si-*PDCD4*. (**B**) The Edu assay for detecting the effect of *PDCD4* on the proliferation of OMECs. (**C**) The proportion of Edu-labeled positive OMECs transfected with pcDNA3.1(+)-*PDCD4* and si-*PDCD4*. ** *p* < 0.01 and * *p* < 0.05.

As shown in Figure 6, when compared with its control group pcDNA3.1(+), overexpressed *PDCD4* remarkably inhibited the viability of OMECs (Figure 6A), as well as the number (Figure 6B) and proportion (Figure 6C) of Edu-labeled positive OMECs, while si-*PDCD4* increased the measurements (Figure 6).

### 2.7. PDCD4 Decreases Triglyceride Content

**Figure 7:**
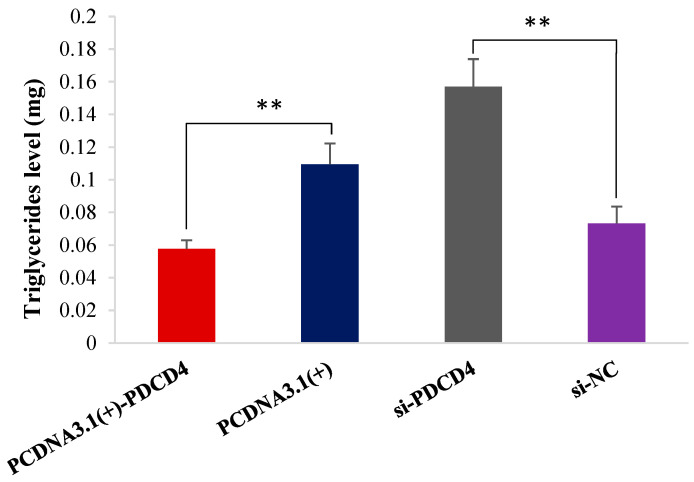
The triglyceride level of ovine mammary epithelial cells (OMECs) transfected with pcDNA3.1(+)-*PDCD4* and si-*PDCD4*. ** *p* < 0.01.

When compared with the NC group-pcDNA3.1(+), pcDNA3.1(+)-*PDCD4* significantly reduced the triglyceride content of OMECs. On the contrary, we tested for an increase in triglyceride content in the cells transfected with si-*PDCD4* (Figure 7).

## 3. Discussion

In this study, miR-21 was found to promote the viability and proliferation of OMECs. This is consistent with other findings for dairy cows. For example, miR-21 was found to promote the proliferation of MECs by targeting insulin-like growth factor-binding protein 5 (*IGFBP5*) [22]. Meanwhile, in other types of cells, miR-21 has also been reported to regulate cell proliferation and apoptosis. Si et al. [23] found that miR-21 inhibited apoptosis by regulating the expression of b-cell lymphoma-2 (*Bcl-2*) in a breast cancer mouse model. Yang et al. [24] found that miR-21-5p promotes cell proliferation in melanoma by targeting cyclin-dependent kinase inhibitor 1C (*CDKN1C*). Given that the proliferation and viability of OMECs are responsible for the lactation performance of ewes, it was therefore inferred that miR-21 may regulate the milk yield and milk composition of ewes.

In view of the fact that miRNA plays its post-transcriptional regulatory roles mainly by complementary binding with its target genes, the target genes of miR-21 were therefore predicted and then validated. Upon the joint analysis of TargetScan v7.2 and miRDB, a total of 161 genes would be predicted to target miR-21. Among all the predicted target genes, *PDCD4* caught our attention. Firstly, *PDCD4* is known as a tumor suppressor gene, and plays an important biological role in cell differentiation and apoptosis [25,26]. Secondly, previous studies have found that miR-21 primarily exerts biological effects in multiple other cells through its target gene *PDCD4*. For example, miR-21 promotes the sustained activation of hepatic stellate cells by down-regulating the expression of *PDCD4* [27]. Xiao et al. [28] found that miR-21 can inhibit cardiomyocytes apoptosis by reducing the expression of *PDCD4*. Subsequently, our dual luciferase reporter assay results confirmed that miR-21 can target the 3′-UTR region of *PDCD4*. The RT-qPCR further found that miR-21 down-regulated the expression level of *PDCD4* in OMECs.

In view of the above discussion, we further investigated the functions of *PDCD4* in the activities of OMECs. It was found that *PDCD4* inhibited the viability, proliferation and milk fat synthesis of OMECs. To the best of our knowledge, this is the first study to report the effects of *PDCD4* on the regulation of the proliferation and viability of MECs in mammals. *PDCD4* is generally considered to be a key regulatory factor for cell apoptosis, and its function is mainly achieved by regulating the PI3K/AKT/GSK-3β pathway [29]. Xia et al. [30] found that the overexpression of *PDCD4* inhibited the PI3K/AKT and JNK signaling pathways, thereby inhibiting cell proliferation. Hua et al. [19] found that the knock-down of ssc-miR-21-5p inhibited the phosphorylation of AKT by targeting *PDCD4*, and eventually led to the inhibition of the proliferation and migration of endometrial epithelial cells. Akt is thought to be a key mediator of signal transduction processes, and its phosphorylation can promote cell survival by inhibiting apoptosis proteins [31,32]. Therefore, we believe that *PDCD4* may regulate the proliferation of OMECs by regulating the AKT signaling pathway. Meanwhile, *PDCD4* has been reported to regulate the proliferation and viability of other type of cells, including human peritoneal mesothelial cells [33], ovarian cancer cells [34] and human breast cancer cells [35]. For example, Hua et al. [19] found that the overexpression of ssc-miR-21-5p can target *PDCD4* to inhibit the apoptosis of endometrial epithelial cell. Fu et al. [36] found that the overexpression of miR-21 inhibited the expression of *PDCD4*, thereby reducing ovarian cell apoptosis. The previous findings were consistent with our results obtained in OMECs.

Milk fat is a determining factor for the nutritional composition of milk. The vast majority of milk fat is composed of triglycerides (98%), besides small amounts of phospholipids, sterols and fat-soluble vitamins [37]. Therefore, the content of triglycerides is crucial for milk composition. Our study found that the overexpression of *PDCD4* reduced the levels of triglycerides in OMECs. A previous study has also found that *PDCD4* inhibited the secretion of triglycerides in dairy goat MECs [38]. The impact of *PDCD4* on lipid metabolism may be achieved by inhibiting regulatory factors of lipid homeostasis [39]. Given that the target relationship of miR-21 with *PDCD4*, and the negative regulatory effects on viability, proliferation and triglyceride levels of OMECs between miR-21 with *PDCD4* affirmed in this study, as well as the effect of *PDCD4* on activities and milk fat synthesis of OMECs, it was concluded that miR-21 promoted the viability, proliferation and milk fat synthesis of OMECs by down-regulating the expression of *PDCD4*.

In addition, miRNA was found to target multiple genes to play its biological role [40]. In this context, miR-21 may regulate the activities and triglyceride levels of OMECs through targeting other target genes in sheep. However, this speculation needs to be further explored. In other MECs, the target relationship of miR-21 with other target genes has been verified. For example, Li et al. [18] found that miR-21 promoted triglyceride yield by targeting *Elovl5* in bovine MECs. Xia et al. [41] also mentioned that miR-21 can increase the triglyceride content in MECs of dairy cows through target functional genes.

## 4. Materials and Methods

### 4.1. Ethics Statement

All procedures involving sheep in this study were strictly conducted in accordance with the Animal Care Committee of Gansu Agricultural University (approval number: GSAU-ETH-AST-2021-027).

### 4.2. Cell Transfection and Real-Time Fluorescence Quantification (RT-qPCR)

According to a previous description [42], OMECs were isolated from the mammary gland tissue of a three-year-old adult Small-tailed Han ewe reared in the Jinzihe Sheep Breeding Company (Tianzhu County, China). And the OMECs were cultured in an incubator at 37 °C with a CO_2_ content of 5%.

The miR-21 mimic (50 nM), miR-21 inhibitor (100 nM), si-*PDCD4* (50 nM) and their corresponding negative controls (NC) (named miR-21 mimic NC, miR-21 inhibitor NC and si-NC) were synthesized by GenePharma Co. Ltd. (Suzhou, China), while the overexpressed vector of *PDCD4* (named pcDNA3.1(+)-*PDCD4*) was synthesized by GENEWIZ Co., Ltd. (Suzhou, China). When the confluence of OMECs cultured in 24-well plates reached 70%~80%, miR-21 mimic, miR-21 inhibitor, si-*PDCD4*, pcDNA3.1(+)-*PDCD4* and their NC were respectively transfected into OMECs using INVI DNA and RNA transfection reagents (Invigentech, Carlsbad, CA, USA). After transfection for 48 h, total RNA from OMECs was extracted using Trizol reagent (Invitrogen, Carlsbad, CA, USA), and its purity was then evaluated using Nanodrop 2000 (Thermo Scientific, Waltham, MA, USA). The extracted RNA was reverse transcribed into cDNA using a HiScript III first strand cDNA synthesis kit (Vazyme, Nanjing, China) and a miRNA 1st Strand cDNA Synthesis Kit (Accurate Biology, Changsha, China), and then RT-qPCR was performed on the Applied Biosystems QuantStudio®6 Flex (Thermo Lifetech, Waltham, MA, USA) using SYBR Premix Ex Taq II (Takara, Dalian, China). An MiRcute miRNA qPCR detection kits (Tianjin, Beijing, China) was used to detect transfection efficiency. The primers used were designed using Primer 5.0 software and are listed in Table 1. The *GAPDH* was used as an internal reference gene to standardize the expression levels of mRNAs, while *U6* and *18sRNA* were chosen as internal references for miRNAs. The relative expression levels of mRNAs and miRNAs were quantified using the 2^−ΔΔCT^ method [43].

### 4.3. Determination of Cell Proliferation and Viability

When the confluence of OMECs reached 70%~80% in the 24-well plates, miR-21 mimic, miR-21 inhibitor, pcDNA3.1(+)-*PDCD4*, si-*PDCD4* and their corresponding NC were transfected into OMECs and then continued to be cultured in a 37 °C and 5% CO_2_ incubator. For the CCK8 assay, 30 μL CCK8 solution (Vazyme, Nanjing, China) was added to each well after 46 h of transfection and then incubated for 2 h. The absorbance of OMECs at 450 nm was measured using Varioskan™ LUX (Thermo Scientific, Waltham, MA, USA). For the EdU assay, 100 mL of 50 mM Edu reagent (Beyotime, Shanghai, China) was added to each well after 44 h of transfection. After incubation for 4 h, the cells were fixed with 4% paraformaldehyde, and then detected using a Cell-Light™ Edu kit (Beyotime, Shanghai, China). Finally, the Edu staining result was observed using an IX73 inverted fluorescence microscope (Olympus, Tokyo, Japan) and then counted using the ImageJ v1.8.0 software (National Institutes of Health, Bethesda, MA, USA).

### 4.4. Validation of the Target Genes for miR-21 Using a Dual-Luciferase Reporter Assay

Two online software programs, TargetScan v7.2 (https://www.targetscan.org/) and miRDB (https://www.mirdb.org), were used to predict the target mRNA binding sites of miR-21 and the predicted results were intersected.

A dual luciferase reporter assay was conducted to verify the target relationship between miR-21 and the predicted target gene *PDCD4*. Briefly, specific primers containing NotI and XhoI restriction sites were designed based on the miR-21 seed sequence and the 3′-UTR sequence information of *PDCD4* (Table 1). The 3′-UTR sequences of *PDCD4* were cloned into the *pmiR-PB-REPORT^TM^* dual luciferase reporter vector to generate a wild-type dual luciferase vector. At the same time, a Mut Express II Fast Mutagenesis Kit (Vazyme, Nanjing, China) was used to generate the mutant sequences of miR-21-5p binding sites on the 3′-UTR, resulting in the generation of a mutate-type dual luciferase vector. Subsequently, when the confluence of HEK293T cells cultured in 12-well plates reached 70% to 80%, the wild-type or mutant-type dual luciferase vectors (3 μg), or miR-21 mimic or miR-21 mimic NC (3 μL), were co-transfected using the INVI DNA & RNA Transfection Reagent^TM^ (Invigentech, Carlsbad, CA, USA). After 48 h of transfection, the dual luciferase reporter assay kit (Promega, Madison, WI, USA) was used to detect the luciferase activity of *PDCD4*, further verifying whether miR-21 has a regulatory effect on the target gene.

Finally, we further investigated the effect of miR-21 on the expression level of the target gene. Specifically, miR-21 mimic, miR-21 mimic NC, miR-21 inhibitor and miR-21 inhibitor NC were transfected into OMECs, as described above. Their transfection efficiency was detected using RT-qPCR. After 48 h, RNA was collected from the cells, and the expression level of *PDCD4* was then detected using a RT-qPCR, with *GAPDH* as a reference gene.

### 4.5. Triglyceride Content Detection

When the fusion rate of ovine MECs cultured in a 12-well plate reached 70% to 80%, the cell triglyceride detection assay kit (Solarbio, Beijing, China) was used to detect the contents of intracellular triglycerides from OMECs independently transfected with miR-21 mimic, miR-21 inhibitor, pcDNA3.1(+)-*PDCD4*, si-*PDCD4* and their corresponding NC.

### 4.6. Statistical Analysis

The statistical analysis of all the data was conducted through a two-tailed independent *t*-test using SPSS 22.0 software, and *p* < 0.05 was considered statistically significant.

## 5. Conclusions

In summary, our results indicate that miR-21 promotes the viability, proliferation and content of triglycerides of OMECs by down-regulating the expression of *PDCD4*. This study contributes to a better understanding of the role of miR-21 in the development and lactation of sheep mammary glands.

## Figures and Tables

**Table 1:** Sequence of primers used in this study.

miRNAs/Genes	Sequence Information		Purpose	Fragment Length and Annealing Temperature
	Forward (5′–3′)	Reverse (5′–3″)		
miR-21	CGCGTAGCTTATCAGACTGATGTTGAC	mRQ 3′ primer	RT-qPCR	60 °C
*PDCD4*	GATGATGACCAGGAGAAC	TCCAACGCTAAGGACACT		200 bp, 58 °C
*18sRNA*	GTGGTGTTGAGGAAAGCAGACA	TGATCACACGTTCCACCTCATC		79 bp, 60 °C
*U6*	ACGGACAGGATTGACAGATT	TCGCTCCACCAACTAAGAA		80 bp, 58 °C
*GAPDH*	ATCTCGCTCCTGGAAGATG	TCGGAGTGAACGGATTCG		173 bp, 58 °C
*PDCD4* (WT)	CCGctcgagAGGGTGTAAAGGAGGGAC	ATTTgcggccgcGAAAGGAGTGGCAGTCAG	Construction of dual luciferase reporter vectors	356 bp, 60 °C
*PDCD4* (Mut)	TTCTCtattcgaACCTTTTGTAAGTGCCATGTTTATG	AAGGTtcgaataGAGAATATCCCACTTAAGAAGTGGTTAC		368 bp, 60 °C

## Data Availability

The data presented in this study are available in the article.
